# Increased Expression of Angiogenic and Inflammatory Proteins in the Vitreous of Patients with Ischemic Central Retinal Vein Occlusion

**DOI:** 10.1371/journal.pone.0126859

**Published:** 2015-05-15

**Authors:** Christoph Ehlken, Bastian Grundel, Daniel Michels, Bernd Junker, Andreas Stahl, Günther Schlunck, Lutz L. Hansen, Nicolas Feltgen, Gottfried Martin, Hansjürgen T. Agostini, Amelie Pielen

**Affiliations:** 1 Klinik für Augenheilkunde, Universitätsklinikum Freiburg, Freiburg, Germany; 2 Hannover Medical School, Eye Hospital, Hannover, Germany; 3 Augenklinik, Universitätsklinikum Göttingen, Göttingen, Germany; International University of Health and Welfare, JAPAN

## Abstract

**Background:**

Central retinal vein occlusion (CRVO) is a common disease characterized by a disrupted retinal blood supply and a high risk of subsequent vision loss due to retinal edema and neovascular disease. This study was designed to assess the concentrations of selected signaling proteins in the vitreous and blood of patients with ischemic CRVO.

**Methods:**

Vitreous and blood samples were collected from patients undergoing surgery for ischemic CRVO (radial optic neurotomy (RON), n = 13), epiretinal gliosis or macular hole (control group, n = 13). Concentrations of 40 different proteins were determined by an ELISA-type antibody microarray.

**Results:**

Expression of proteins enriched in the vitreous (CCL2, IGFBP2, MMP10, HGF, TNFRSF11B (OPG)) was localized by immunohistochemistry in eyes of patients with severe ischemic CRVO followed by secondary glaucoma. Vitreal expression levels were higher in CRVO patients than in the control group (CRVO / control; p < 0.05) for ADIPOQ (13.6), ANGPT2 (20.5), CCL2 (MCP1) (3.2), HGF (4.7), IFNG (13.9), IGFBP1 (14.7), IGFBP2 (1.8), IGFBP3 (4.1), IGFBP4 (1.7), IL6 (10.8), LEP (3.4), MMP3 (4.3), MMP9 (3.6), MMP10 (5.4), PPBP (CXCL7 or NAP2) (11.8), TIMP4 (3.8), and VEGFA (85.3). In CRVO patients, vitreal levels of CCL2 (4.2), HGF (23.3), IGFBP2 (1.23), MMP10 (2.47), TNFRSF11B (2.96), and VEGFA (29.2) were higher than the blood levels (vitreous / blood, p < 0.05). Expression of CCL2, IGFBP2, MMP10, HGF, and TNFRSF11B was preferentially localized to the retina and the retinal pigment epithelium (RPE).

**Conclusion:**

Proteins related to hypoxia, angiogenesis, and inflammation were significantly elevated in the vitreous of CRVO patients. Moreover, some markers known to indicate atherosclerosis may be related to a basic vascular disease underlying RVO. This would imply that local therapeutic targeting might not be sufficient for a long term therapy in a systemic disease but hypothetically reduce local changes as an initial therapeutic approach.

## Introduction

Retinal vein occlusion is the second most common vascular eye disease and causes vision loss due to macular edema, retinal bleeding and ischemia [1]. The worldwide prevalence is estimated at 1:1250 [2]. Central retinal vein occlusion (CRVO) is less frequent than branch retinal vein occlusion (BRVO) but results in greater retinal damage.

Visual acuity (VA) prognosis in CRVO is significantly improved by treatment of macular edema either with intravitreal steroids or anti-VEGF therapeutics that address inflammatory and VEGF-driven ocular changes [3]. Intravitreal anti-VEGF treatment leads to significant visual gain of 15 letters or more in up to 60% of the patients (47% ranibizumab [4], 55% aflibercept [5], 60% bevacizumab [6]) at one year. However, final VA of ≥ 20/40, sufficient to allow for driving and reading, is only reached in every second patient (47% ranibizumab [4]). This underlines the need for a detailed characterization of risk factors and further improvement of treatment strategies.

Known risk factors for RVO are advanced age [1], glaucoma and systemic diseases, especially components of the metabolic syndrome such as diabetes mellitus, hypertension and hyperlipidemia [7]. Regarding diabetes, patients with end-organ damage from diabetes have a significantly increased risk of CRVO, while those without do not [7]. Hyperlipidemia leads to atherosclerosis, which represents a later state of the disease. Atherosclerosis of the central retinal artery was found in association with CRVO [8]. The hypothesis that atherosclerosis is associated with a higher risk of CRVO is supported by the finding that history of stroke and peripheral arterial disease are associated with higher incidence of CRVO [7,9,10].

Inflammatory cytokines, chemokines and neurotrophic factors have been investigated in the vitreous of patients with retinal vascular diseases due to diabetes or retinal vein occlusion. VEGF is upon the most investigated as anti-VEGF is implemented in therapy [3,11]. Elevated levels of inflammatory immune mediators such as IL-6, IL-8, CCL2 were reported in central and branch RVO, diabetic macular edema, proliferative diabetic retinopathy and retinal detachment [12]. Others found significantly higher levels of IL-1β, IL-2, IL-5, IL-8, IL-9, IL-10, IL-12, IL-13, CCL11, G-CSF, IFN-γ, CXCL10, CCL2, CCL4, TNF, and VEGF specifically in CRVO [13]. An association between the expression of inflammatory factors and the severity of macular edema was observed in CRVO [14]. Levels of VEGF, IL-6, sICAM-1 and PEDF correlated independently with vascular permeability. These factors were higher in CRVO than in controls, higher in ischemic versus non-ischemic CRVO and correlated with macular edema in optic coherence tomography [14].

Analysis of plasma levels of atherosclerotic and thrombophilic risk factors demonstrated that arterial hypertension, hypercholesterolemia, hyperhomocysteinemia and elevated factor VIII were associated with an increased risk for ischemic versus non-ischemic CRVO [15]. We set out to simultaneously investigate the expression of 40 proteins associated with inflammation, hypoxia, angiogenesis and atherosclerosis in vitreous and blood samples of patients undergoing RON (radial optic neurotomy) for clinically defined ischemic CRVO and compared it to a control group of patients receiving surgery for epiretinal gliosis or a macular hole. Criteria for the selection of the proteins to measure were solubility in the cytoplasm (as we did not expect cells or cell membranes in the vitreous), a context with angiogenesis and inflammation, and availability from the provider of the array. Our data suggest that distinct chemokines (CCL2) and growth factors (HGF) may represent valuable targets for novel therapeutic approaches to treat or prevent ischemic complications in CRVO patients. The observations also support epidemiologic data regarding risk factors such as atherosclerosis.

## Materials and Methods

### Ethics statement

All patients gave their written informed consent prior to their inclusion in the study. The study was registered as experimental laboratory investigation at the Center of Clinical Trials and approved by the Institutional Review Board of the University Freiburg (No 215/08) and performed in accordance with the IRB’s requirements, with the ethical standards laid down in the 1964 Declaration of Helsinki and with the federal laws in Germany.

### Patients and study design

Patients with ischemic CRVO were recruited between 2005 and 2006. At the time of sample acquisition, radial optic neurotomy was thought a valuable surgical approach for ischemic CRVO. However, this technique did not fulfil expectations [16]. In recent years, intravitreal anti-VEGF treatment has been introduced to treat macular edema secondary to CRVO. It is currently the new standard of treatment for either non-ischemic and ischemic CRVO and surgical approaches are left to rare severe cases. CRVO patients with ischemic occlusive disease, indicated either by nonperfusion in fluorescence angiography (> 10 disc diameters), visual acuity > 1.0 log MAR, and/or clinical findings such as dark hemorrhages, a high number of cotton wool spots, or massive leakage of the vessels and papilledema [17], were selected for vitrectomy and radial optic neurotomy (n = 13). Duration of CRVO was defined as time from onset of symptoms until surgery. Neovascularizations of the iris were found in 2/13 patients. Control specimens were collected from 13 patients undergoing vitrectomy for macular pucker and macular hole. Patient data is presented in Table 1. CRVO patients did not show differences compared to control regarding age and the risk factors arterial hypertension, diabetes, and history of stroke. Significantly more CRVO patients presented with hyperlipidemia, history of smoking, glaucoma and use of anticoagulants (aspirin or phenprocoumon), indicating a higher prevalence of cardiovascular diseases known as risk factors for CRVO.

**Table 1:** Patient characteristics.

	CRVO	Control
**Number of patients (male)**	13 (5)	13 (4)
**Age (mean ± SD, years)**	74.6 ± 9.7	69.5 ± 9.8
**Duration of CRVO (weeks)**	9.4 ± 5.9	not applicable
**Neovascularisation of the iris (at time of surgery)**	2/13 (15%)	0 (0%)
**Neovascularisation of the disc or elsewhere in the retina**	0 (0%)	0 (0%)
**Mean visual acuity (log MAR +/- SD)**	1.6 +/- 0.48	not applicable
**Risk factors for CRVO (%):**		
**Glaucoma**	6/13 (46%)	0 (0%)
**Arterial hypertension**	10/13 (77%)	11/13 (85%)
**Diabetes**	0 (0%)	0 (0%)
**Hyperlipidemia**	2/13 (15%)	0 (0%)
**History of stroke**	1/13 (8%)	1/13 (8%)
**Smoking**	2/13 (15%)	0 (0%)
**Anticoagulation**	5/ 13 aspirin 2/ 13 marcumar	2/ 13 aspirin 0/ 13 marcumar

SD = standard deviation

BCVA = best corrected visual acuity

CRVO = central retinal vein occlusion

Control = epiretinal gliosis or macular hole.

A standard 3-port vitrectomy was performed during surgery. Sample acquisition was achieved as the first step of the surgery avoiding dilution by the infusion. Depending on clinical findings, additional procedures such as laser photocoagulation, intravitreal administration of triamcinolone or bevacizumab could be included at the end of surgery. Samples (200–400 μl each) were immediately stored at -80°C until further investigation.

Patients with other proliferative eye diseases, such as uveitis or diabetic retinopathy, or patients with intraocular surgery within the last 6 months, or history of vitrectomy, were excluded from the study.

### Measurement of proteins

Concentrations of various proteins from vitreous and blood samples were measured with an ELISA-type antibody microarray (Quantibody, Raybiotech Inc., Norcross, GA) following the manufacturer’s instructions. Antibodies for each protein were arrayed in quadruplicates per array. 80 μl of vitreous or blood was used for each sample. The detection antibodies were labelled with biotin which was detected with Alexa Fluor 555-conjugated streptavidin. The signals were read with a G2565 microarray reader (Agilent Technologies, Santa Clara, CA). TM4 Spotfinder (http://www.tm4.org, [18]) was used for quantification of the spots. The concentrations of the proteins were calculated from the median intensities of the spots using standard curves obtained with a mix of the 40 peptide standards. Detection limits were calculated from the standard curves with DINTEST (http://www.luiw.ethz.ch/computer/software/) according to DIN 32645. Protein concentrations were determined using the Bradford assay with BSA as a standard [19] as the BCA (Bicinchoninic acid) test resulted in erroneously high values if the proteins were not precipitated.

Mean concentrations for CRVO and control groups were compared by nonparametric comparisons (R package nparcomp, http://www.r-project.org/) with Tukey’s correction for multiple comparisons. Correlation between concentrations and the time after CRVO was determined by the Pearson product-moment correlation coefficient. p < 0.05 was considered significant. Correlations among proteins as well as among patients were tested in R with corr (psych package) using the Spearman coefficient and Holm adjustment for multiple comparison. Biochemical pathways were analyzed by enrichment analysis (EnrichmentBrowser and gage in Bioconductor) against the KEGG pathways database (http://www.genome.jp/kegg/) and the gene ontology database (http://geneontology.org/) for the CRVO patients and vitreous samples with 24 genes showing expression above background. A complete list of factors tested, gene symbol, gene ID and gene name is provided as supplementary S1 Table.

### Immunohistochemistry

Enucleated eyes from two female patients (88 and 61 years old) with severe ischemic CRVO followed by neovascular glaucoma were investigated for expression of the proteins that showed higher concentrations in the vitreous than in blood. Sections (5 μm) of paraffin embedded eyes were dewaxed and demasked for 20 min in 100 mM sodium citrate, pH 6.0, in a steamer. After transfer to TBST (50 mM Tris / HCl pH 7.6, 0.9% NaCl, 0.02% Tween 20), sections were blocked with Ultra V Block (Lab Vision at medac GmbH, Wedel, Germany) and incubated with antibodies as listed in Table 2 for 3 h. After washing in TBST, an AP-labelled goat anti-mouse secondary antibody (A3562, Sigma-Aldrich, Taufkirchen, Germany) was applied for 1 h, or a biotin-labelled goat anti-rabbit secondary antibody (71-00-30, KPL, Gaithersburg, MD, USA) was applied for 1 h followed by streptavidin-coupled AP (71-00-45, KPL) for 1 h. Slides were washed and AP was made visible by the Vector Red AP Substrate Kit I (SK-5100, Vector Labs at Axxora, Lörrach, Germany). Sections were counter-stained with hematoxylin.

**Table 2:** Antibodies used for immunohistochemistry.

Antibody against	Company	Product No.	Type	Host	Dilution
**ADIPOQ**	Acris	SP2182P	Polyclonal	rabbit	1:50
**CCL2**	Acris	PP1044P1	Polyclonal	rabbit	1:400
**COL IV**	Abcam	ab6586	Polyclonal	rabbit	1:500
**GFAP**	Dako	Z0334	Polyclonal	rabbit	1:1000
**HGF**	Aviva	ARP44317_P050	Polyclonal	rabbit	1:400
**IBA1**	Wako	019–19741	Polyclonal	rabbit	1:500
**IGFBP2**	GeneTex	GTX113471	Polyclonal	rabbit	1:400
**MMP10**	Abcam	ab38930	Polyclonal	rabbit	1:1000
**PPBP**	PeproTech	500-P03	Polyclonal	rabbit	1:50
**TNFRSF11B**	Acris	SM7070P	Monoclonal	mouse	1:50

COL IV: collagen IV

GFAP: glial fibrillary acidic protein

IBA1: allograft inflammatory factor 1 (AIF1).

## Results

### Concentration of various proteins in the vitreous or blood

The blood concentrations of the proteins investigated in this study were similar in CRVO patients compared to control patients (range of the ratios between 0.22 and 1.93, median 1.04, Table 3). In contrast, the concentration of total protein in the vitreous of CRVO patients was 6.4 fold elevated compared to that of control patients (Table 3). The vitreous concentrations of proteins ADIPOQ, ANGPT2, CCL2, HGF, IFNG, IGFBP1, IGFBP2, IGFBP3, IGFBP4, IL6, LEP, MMP3, MMP9, MMP10, PPBP, TIMP4, and VEGFA were elevated in CRVO patients compared to control patients (range of the ratios between 0.75 and 85.3, median 1.82, Table 3). Proteins like FGF6, FGF7, MMP1, TIMP1, and TIMP2 did not show enhanced vitreous concentrations in CRVO patients compared to controls. This indicates that there was not only a break-down of the blood retina barrier but also a local production within the eye or a selective transport of proteins. The total increase of the proteins measured in this study was 1.1 μg/ml in the vitreous (mainly contributed by PPBP), while the increase in total vitreal protein was 2.8 mg/ml indicating a 2500 fold impact of blood retina barrier break-down as compared to ocular protein expression. Taking into account the ocular expression of proteins not measured in this study, the factor will be somewhat smaller than 2500.

**Table 3:** Concentration of various factors in vitreous fluid and blood serum of CRVO patients and controls.

		CRVO			Control			Vitreous		Correlation with time after CRVO	
Gene	Unit	Blood ± SD	Vitreous ± SD	V / B	Blood ± SD	Vitreous ± SD	V / B	CRVO / Control	Detection limit	Blood	Vitreous
**ADIPOQ**	ng/ml	151 ± 23	61 ± 24	0.40 *	135 ± 25	4.5 ± 3.9	0.03 *	**13.6** *	1.0	-0.22	-0.28
**ANGPT2**	pg/ml	1268 ± 1258	1619 ± 1120	1.28	1120 ± 554	79 ± 26	0.07 *	**20.5** *	63	0.01	-0.33
**CCL2**	pg/ml	193 ± 85	809 ± 118	**4.20** *	218 ± 92	252 ± 113	1.15	**3.2** *	17	-0.13	-0.55 *
**HGF**	ng/ml	0.50 ± 0.26	11.7 ± 6.4	**23.3** *	0.55 ± 0.32	2.5 ± 1.5	**4.55** *	**4.7** *	0.13	-0.33	-0.28
**IFNG**	pg/ml	725 ± 273	97 ± 110	0.13 *	998 ± 559	7 ± 12	0.01 *	**13.9** *	43	-0.21	-0.33
**IGFBP1**	ng/ml	7.8 ± 2.8	3.0 ± 1.8	0.38 *	6.1 ± 1.7	0.20 ± 0.12	0.03 *	**14.7** *	0.13	0.38	-0.06
**IGFBP2**	ng/ml	9.3 ± 1.5	11.4 ± 1.6	**1.23** *	8.8 ± 1.1	6.4 ± 2.8	0.73 *	**1.8** *	0.44	-0.09	-0.41
**IGFBP3**	ng/ml	51 ± 14	6.8 ± 3.4	0.13 *	36 ± 11	1.7 ± 0.69	0.05 *	**4.1** *	1.4	-0.08	-0.29
**IGFBP4**	ng/ml	68 ± 24	9.4 ± 2.6	0.14 *	50 ± 23	5.4 ± 2.2	0.11 *	**1.7** *	4.0	0.22	-0.28
**IL6**	pg/ml	94 ± 31	43 ± 38	0.46 *	118 ± 60	4 ± 3	0.03 *	10.8 *	6	-0.19	-0.42
**LEP**	ng/ml	10 ± 10	0.98 ± 0.96	0.10 *	33 ± 41	0.29 ± 0.12	0.01 *	**3.4** *	0.40	0.26	0.67 *
**MMP3**	ng/ml	12.0 ± 6.4	1.02 ± 0.62	0.08 *	9.3 ± 5.1	0.24 ± 0.29	0.03 *	**4.3** *	0.40	-0.36	-0.40
**MMP9**	pg/ml	13039 ± 6806	142 ± 63	0.01 *	11886 ± 7446	39 ± 14	0.00 *	**3.6** *	43	-0.47	-0.29
**MMP10**	pg/ml	198 ± 306	488 ± 350	**2.47** *	339 ± 236	90 ± 58	0.26 *	**5.4** *	47	-0.42	-0.27
**PPBP**	ng/ml	4867 ± 1522	1105 ± 442	0.23 *	3091 ± 1488	94 ± 80	0.03 *	**11.8** *	9.0	0.03	-0.73 *
**TIMP4**	ng/ml	3.9 ± 1.7	1.1 ± 0.39	0.28 *	2.70 ± 0.87	0.29 ± 0.10	0.11 *	**3.8** *	0.17	0.46	-0.43
**TNFRSF11B**	ng/ml	1.42 ± 0.76	4.2 ± 1.6	**2.96** *	2.1 ± 2.1	2.7 ± 1.7	1.30	1.5	0.19	0.09	0.40
**VEGFA**	pg/ml	202 ± 278	5883 ± 4503	**29.2** *	398 ± 413	69 ± 27	0.17 *	**85.3** *	136	-0.24	-0.46
*ANGPT1*	ng/ml	13 ± 12	0 ± 0	0.00 *	25 ± 15	0 ± 0	0.00 *	-	1.5	-0.44	-
*CCL7*	pg/ml	74 ± 22	4 ± 2	0.06 *	55 ± 29	2 ± 1	0.04 *	2.0 *	14	0.17	-0.38
*CXCL11*	pg/ml	70 ± 38	3 ± 2	0.04 *	49 ± 29	4 ± 3	0.08 *	0.75	6	0.25	0.09
*EGF*	pg/ml	854 ± 721	0 ± 1	0.00 *	1867 ± 1246	0 ± 1	0.00 *	-	12	-0.41	0.11
*FGF2*	pg/ml	361 ± 160	139 ± 26	0.39 *	338 ± 129	151 ± 42	0.45 *	0.92	209	-0.03	0.69 *
*IGF1*	pg/ml	3963 ± 1873	85 ± 167	0.02 *	3417 ± 2076	97 ± 236	0.03 *	0.88	2078	0.06	-0.44
*IGFBP5*	ng/ml	6.9 ± 4.1	3.0 ± 3.7	0.43	6.6 ± 5.7	1.6 ± 1.2	0.24 *	1.9	3.8	0.17	-0.11
*IL1B*	pg/ml	14 ± 6	7 ± 2	0.51 *	13 ± 7	8 ± 2	0.63	0.88	11	0.17	0.26
*IL4*	pg/ml	47 ± 23	10 ± 6	0.21 *	46 ± 29	7 ± 4	0.15 *	1.4	10	-0.17	-0.36
*IL13*	pg/ml	53 ± 18	7 ± 8	0.13 *	81 ± 51	0 ± 0	0.00 *	- *	13	-0.41	-0.21
*IL18BP*	pg/ml	592 ± 263	110 ± 56	0.19 *	584 ± 290	65 ± 28	0.11 *	1.7 *	133	-0.24	-0.49
*MMP2*	pg/ml	838 ± 350	142 ± 52	0.17 *	453 ± 520	150 ± 96	0.33	0.95	179	0.04	0.30
*MMP8*	pg/ml	87 ± 95	9 ± 14	0.10 *	76 ± 78	4 ± 5	0.05 *	2.25	22	-0.30	-0.34
*TNF*	pg/ml	197 ± 117	11 ± 33	0.06 *	887 ± 971	0 ± 0	0.00 *	-	65	-0.31	-0.20
*TNFRSF18*	pg/ml	210 ± 132	16 ± 9	0.07 *	263 ± 140	11 ± 9	0.04 *	1.5	76	-0.36	-0.34
FGF6	pg/ml	174 ± 99	43 ± 6	0.25 *	166 ± 75	40 ± 7	0.24 *	1.08	38	-0.02	0.46
FGF7	pg/ml	143 ± 90	23 ± 8	0.16 *	198 ± 181	25 ± 12	0.13 *	0.92	15	-0.24	-0.47
MMP1	ng/ml	1.95 ± 2.6	0.33 ± 0.10	0.17 *	4.7 ± 4.5	0.27 ± 0.12	0.06 *	1.2	0.20	-0.45	0.23
TIMP1	ng/ml	34 ± 19	5.4 ± 1.6	0.16 *	33 ± 18	4.4 ± 2.5	0.13 *	1.2	0.22	-0.47	-0.33
TIMP2	ng/ml	15.0 ± 5.4	9.6 ± 1.4	0.64 *	7.7 ± 3.1	8.0 ± 2.6	1.03	1.2	0.32	-0.39	-0.40
**Protein**	mg/ml	46 ± 12	3.3 ± 1.7	0.07 *	56 ± 12	0.51 ± 0.58	0.01 *	6.4 *	0.48		

*significant difference as determined by nonparametric comparisons (p < 0.05)

Criteria for selected factors labeled in bold: vitreous concentration higher than blood values; or vitreous concentration of CRVO and control significantly different, and at least one value for vitreous above the detection limit. Italic factors: Vitreous values below detection limit.

All values for FGF4 and MMP13 were below the detection limit, and the values for concentrations of ANGPT1, CCL7, CXCL11, EGF, FGF2, IGF1, IGFBP5, IL1B, IL4, IL13, IL18BP, MMP2, MMP8, TNF, and TNFRSF18 in the vitreous of both CRVO patients and controls were below the detection limit.

The column “Correlation with time after occlusion” shows the Pearson product-moment correlation coefficient that is a measure of the linear correlation between the protein concentration in the vitreous or blood and the time after occlusion. * indicates statistical significance (p < 0.05). Note that the significance of LEP is lost if the highest value is omitted. The time after occlusion is the time between the CRVO and vitrectomy.

SD = standard deviation

V / B = vitreous / blood

CRVO = central retinal vein occlusion.

In CRVO patients, most of the proteins investigated had significantly higher concentrations in blood than in the vitreous. However, protein concentrations (ratio vitreous / blood) of CCL2 (4.2), HGF (23.3), IGFBP2 (1.23), MMP10 (2.47), TNFRSF11B (2.96), and VEGFA (29.2) were significantly higher in the vitreous than in the blood of the same patient (p < 0.05, Table 3). This indicates that the proteins showing higher concentrations in the vitreous were, at least partially, produced within the eye or actively transported there. In control patients, only HGF showed significantly higher concentrations in the vitreous than in blood. Statistical analysis of the correlations among proteins or patients were not conclusive, most probably because of the small number of proteins and patients. The same was true for the biochemical pathway analyses.

### Dependence of the protein concentrations from the time after occlusion

**Fig 1:**
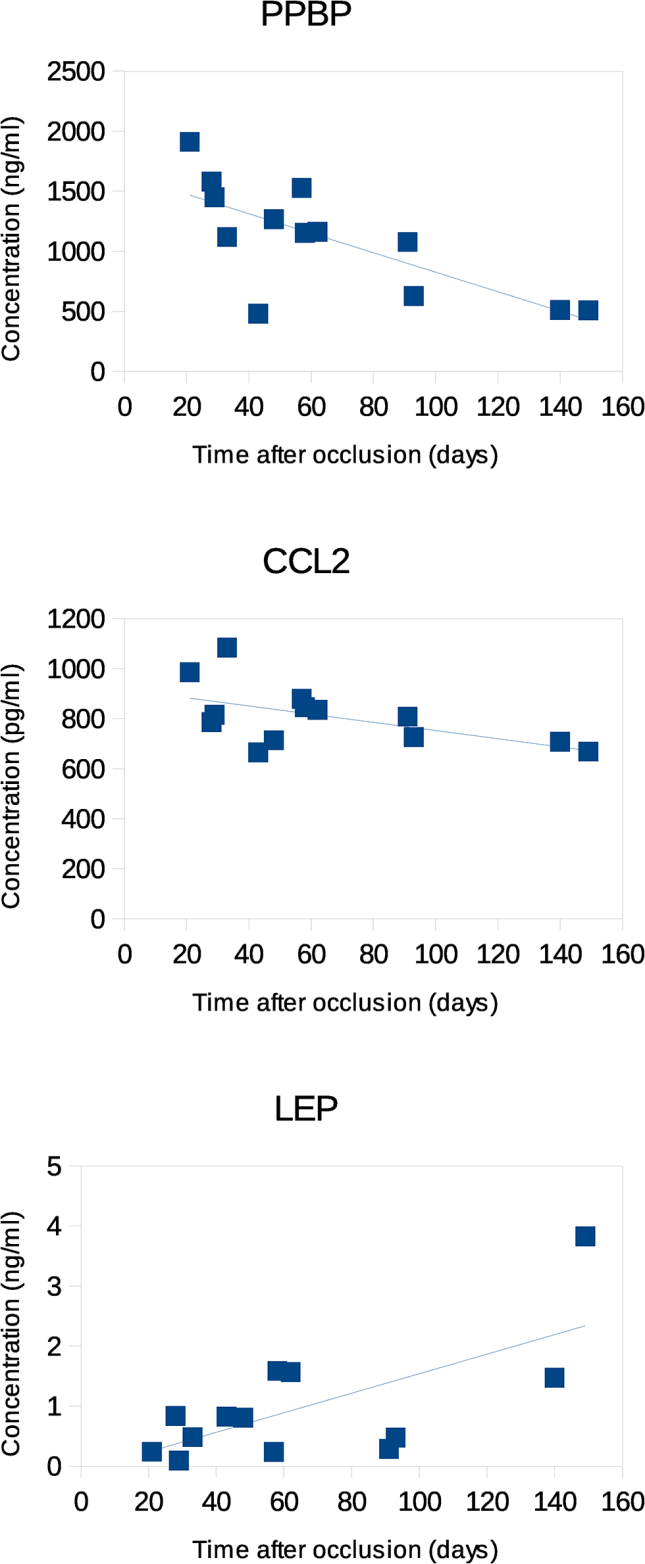
Correlation of the vitreal concentration of PPBP, CCL2, and LEP with the time after occlusion (time between onset of symptoms due to CRVO and surgery). Each data point represents a pair of data from an individual patient. Note that the significance of LEP is lost if the highest value is omitted.

The time between the onset of symptoms due to CRVO and the time point at which the vitreous specimen was taken was different for each patient (time after occlusion, mean: 9.4 ± 5.9 weeks). As surgery was performed once in every patient, specimens could not be taken at different time points which limits the interpretation of the results. We compared the concentrations of the proteins measured to the time after occlusion (Table 3). PPBP (-0.73, p<0.05) and CCL2 (-0.55, p<0.05) showed a negative correlation (Fig 1) that may reflect an increased selective permeability for certain small proteins or an increased inflammatory or angiogenic state shortly after CRVO that is repaired with time. Similar tendencies, though not statistically significant, were found for IGFBP2, IL6, MMP3, TIMP4, and VEGFA. In contrast, LEP showed a positive correlation (0.67, p<0.05).

### Localization of selected proteins in the human eye

**Fig 2:**
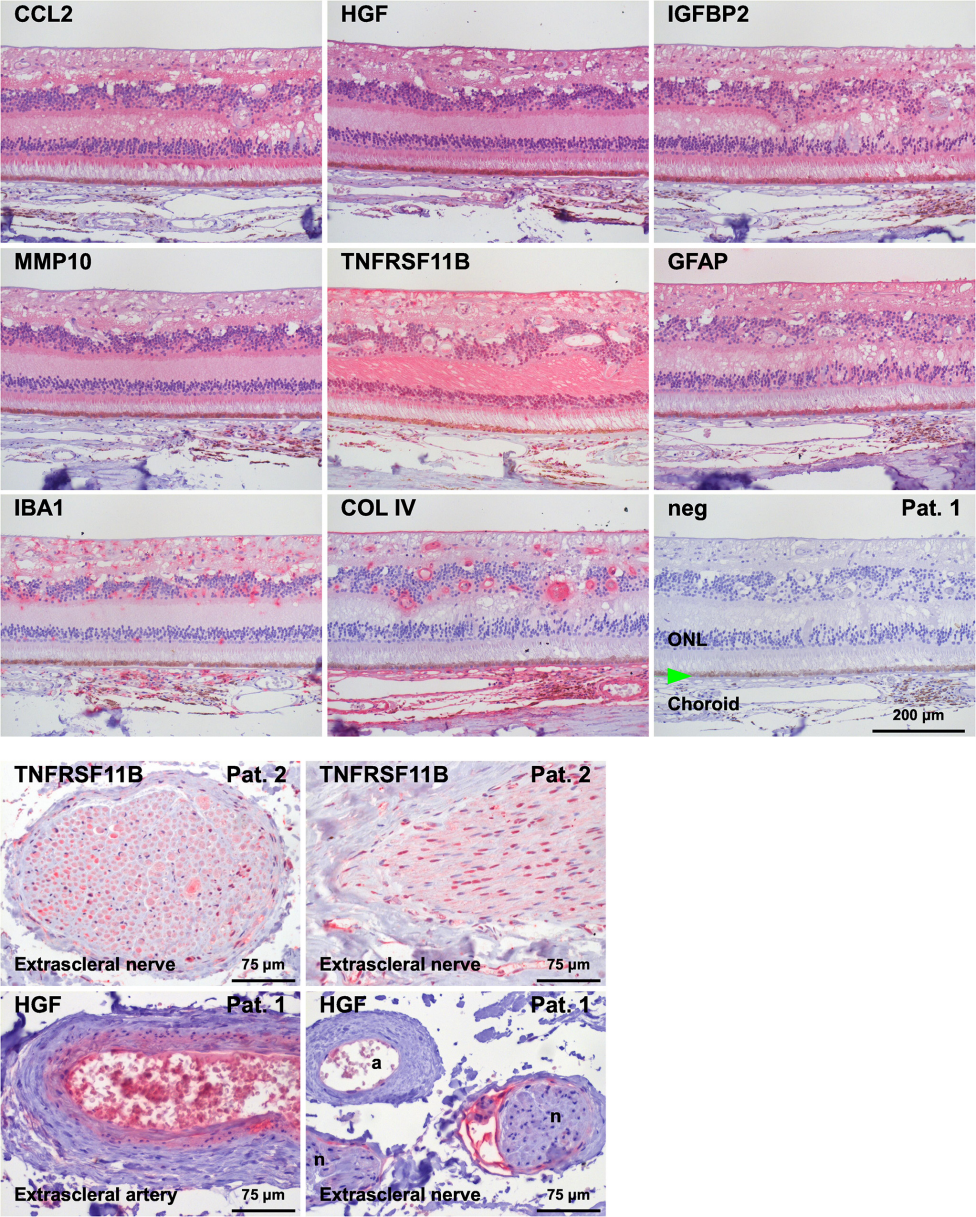
Immunohistochemical staining (alkaline phosphatase, red; blue counter staining: hematoxylin) for CCL2, HGF, IGFBP2, MMP10, and TNFRSF11B in ocular samples of various patients. These factors were found preferentially in the retina and additionally in nerves and in the RPE. TNFRSF11B was found in the axons and some nuclei of extrascleral nerves. HGF was additionally found in the endothelium and media of some but not all extrascleral vessels. GFAP (glia cell marker), IBA1 (migroglia and macrophage marker, and COL IV (basement membrane marker, e.g. in the basement membrane of vessels and in the membrana limitans interna) are shown for comparison. Note that GFAP is not expressed in the outer segments of the photoreceptors as is the case for CCL2, HGF, IGFBP2, MMP10, and TNFRSF11B. neg: negative control without primary antibody, a: artery, n: nerve, ONL: outer nuclear layer, green arrow: RPE.

Ocular localization of the proteins that showed significantly higher expression in the vitreous than in blood (CCL2, IGFBP2, MMP10, HGF, TNFRSF11B) was investigated in histological specimens of eyes from patients with painful blindness due to secondary glaucoma after CRVO (Fig 2). Staining for all these factors was found preferentially in the retina and in the retinal pigment epithelium (RPE) but to a much lesser extent in the optic nerve head or extrascleral nerves. Staining intensity was higher in ocular areas affected with inflammation. HGF was additionally found in the endothelium and media of some but not all extrascleral vessels. Staining for GFAP (glial marker), IBA1 (microglial and macrophage marker), and COL IV (marker for basement membranes of vessels) was used for comparison.

## Discussion

Retinal vein occlusion and subsequent ischemia are followed by the release of cytokines, growth factors and enzymes which contribute to severe vision loss due to retinal edema and neovascularization. Previous studies in patients suffering from retinal vein occlusions detected a range of proteins in the vitreous [20,21]. Vitreal VEGFA concentrations were also determined earlier [11,22,23] and used as a reference in this study. In contrast to previous reports, we had the opportunity to assess distinct protein levels in the vitreous as well as in blood samples of patients following ischemic CRVO and compare them to unrelated controls. This allowed us to further characterize CRVO-specific changes in vitreal protein expression patterns and to gather evidence for ischemia-induced localized expression of distinct proteins as opposed to a release from blood.

Most of the proteins that were found to be more prevalent in the vitreous of CRVO patients than in controls appear to be strongly related to hypoxia, inflammation or angiogenesis. VEGFA, ADIPOQ, ANGPT2, CCL2, IGFBP1, or LEP share a common hypoxia-response element (HRE) at their promoter or intron [24–27] indicating that they are regulated by HIF1A or HIF2A. In addition, IGFBP2 and IGFBP3 are known to be up-regulated upon hypoxia [28,29], but it is currently unclear if they are upregulated by HIF or by one of his target genes. Three of these factors (VEGFA, CCL2 and IGFBP2) showed significantly higher levels in the vitreous than in the blood of CRVO patients. These data are clearly consistent with the activation of hypoxia-induced gene networks and a localized intraocular expression of specific proteins due to a hypoxic state.

The transcription factor NFKB is upregulated by hypoxia. It has a central role in inflammation as it induces IFNG [30] and IL6 [31]. Both were detected in the vitreous of CRVO patients and significantly increased compared to controls. In atherosclerosis, CCL2 (also named MCP-1) is involved in initial steps of inflammation by attracting monocytes, T-cells and dendritic cells [32,33]. These data strongly support the notion that CRVO is inducing an inflammatory response in the vitreous.

VEGFA, HGF, MMP3 and MMP9 share common ETS1 binding sites in their promoter region [34–36]. The transcription factor ETS1 is expressed in endothelial cells and upregulates genes involved in angiogenesis. ETS1 itself is induced by angiogenic factors like VEGF, HGF, or FGF2 resulting in a positive feed-back loop [37,38]. Moreover, expression of ETS1 is induced by HIF1A [39] linking angiogenesis to hypoxia in addition to the up-regulation of VEGFA by HIF. The metalloprotease MMP10 is induced by the transcription factor MEF2 in response to VEGFA [40,41]. This indicates that significant vitreal levels of angiogenic signaling factors are present in ischemic CRVO before neovascular changes are clinically apparent. In addition, these findings point towards a set of angiogenic target proteins including HGF and selected MMPs which may be amenable to pharmacological intervention.

HGF was found to be increased in vitreous samples of patients with proliferative diabetic retinopathy and was higher in vitreous than in blood similar to our results [42]. The intraocular expression of CCL2, HGF, IGFBP2, MMP10, and TNFRSF11B was confirmed by immunohistochemistry in eyes of patients with secondary glaucoma after RVO. This validates some of our earlier results and provides strong evidence that these proteins are expressed in ocular tissues. For most of them, expression within the eye has been reported earlier: CCL2, HGF [43,44], IGFBP2, MMP10, TNFRSF11B, and VEGFA [45] demonstrating that at least one cell type in the eye can produce these proteins under certain conditions.

Current ocular treatment is focused on anti-VEGF agents and anti-inflammatory steroids. Our data may add therapeutic targets to improve current anti-VEGF therapy in ischemic CRVO. Further investigation in the factors associated with hypoxia, inflammation and angiogenesis in ischemic CRVO may also lead to new therapeutic approaches to prevent conversion from non-ischemic to ischemic CRVO.

We also asked, whether our limited sample reflects known risk factors for retinal vein occlusion such as metabolic syndrome (diabetes, hypertension, hyperlipidemia (> 1 factor)), atherosclerosis of central retinal artery, history of stroke, and peripheral artery disease. More CRVO patients presented with one or more risk factors compared to controls (most pronounced differences in hyperlipidemia, smoking and use of anticoagulation). This is in line with previous findings: Analysis of plasma levels of atherosclerotic and thrombophilic risk factors demonstrated that arterial hypertension, hypercholesterolemia, hyperhomocysteinemia, elevated factor VIII were associated with an increased risk for ischemic versus non ischemic CRVO [15]. Our findings stress the need for careful work-up of ischemic CRVO patients to detect risk factors and adequately treat all the patient’s diseases.

Pathophysiology of CRVO is not yet completely clear, but it is agreed that atherosclerotic changes of the retinal arteries contribute to the disease [46]. With regard to the vitreal proteins detected in CRVO patients, TNFRSF11B is a marker of atherosclerosis [47], though its pathophysiological role is yet unclear [48]. Similarly, serum concentration of TIMP4 is increased in systemic sclerosis [49]. TIMP4 is the major MMP inhibitor in platelets and is released upon platelet aggregation induced by collagen and thrombin [50,51]. MMP10 is upregulated by thrombin in endothelial cells and enhances fibrinolysis [52,53]. PPBP is expressed upon platelet activation during thrombus formation [54]. PPBP expression is induced by MMP3 [55]. Both MMP9 and CCL2 are associated with atherosclerosis [56] where CCL2 attracts monocytes that mature into macrophages and produce MMP9. This cleaves components of the extracellular matrix within the atherosclerotic plaques. Thus, several of the vitreal proteins we detected are consistent with an atherosclerotic phenotype. Since data on the vitreal protein expression patterns preceding the retinal vein occlusion are not available, it remains challenging to dissect which vitreal proteins reflect an underlying chronic condition rather than an acute occlusion response.

In summary, ischemic CRVO is characterized by increased vitreal levels of a distinct set of proteins, some of them locally expressed, which may serve as targets for novel therapeutic approaches to augment current anti-inflammatory and anti-angiogenic treatments.

## Supporting Information

S1 Table Factors tested. V / B = vitreous / blood. CRVO = central retinal vein occlusion. Corr. = correlation.
(DOCX)
