# Supplementary material for: Increased Expression of Angiogenic and Inflammatory Proteins in the Vitreous of Patients with Ischemic Central Retinal Vein Occlusion
Source: PLoS One. 2015 May 15;10(5):e0126859. doi: 10.1371/journal.pone.0126859 (PMC4433200; doi:10.1371/journal.pone.0126859)
Supplement: S1 Table — V / B = vitreous / blood. CRVO = central retinal vein occlusion. Corr. = correlation. (DOCX) [file pone.0126859.s001.docx]

**S1 Table:** Factors tested

|  |  |  | **p values** | | | | |
| --- | --- | --- | --- | --- | --- | --- | --- |
| **Gene Symbol** | **Gene ID** | **Gene Name** | **CRVO (V / B)** | **Control (V / B)** | **Vitreous (CRVO / Control)** | **Corr. Blood** | **Corr. Vitreous** |
| ADIPOQ | 9370 | adiponectin | 0,0000 | 0,0000 | 0,0000 | 0,4801 | 0,3479 |
| ANGPT1 | 284 | angiopoietin 1 | 0,0000 | 0,0000 | 1,0000 | 0,1331 |  |
| ANGPT2 | 285 | angiopoietin 2 | 0,8866 | 0,0000 | 0,0000 | 0,9799 | 0,2716 |
| CCL2 | 6347 | chemokine (C-C motif) ligand 2, MCP1 | 0,0000 | 0,9740 | 0,0000 | 0,6763 | 0,0502 |
| CCL7 | 6354 | chemokine (C-C motif) ligand 7, MCP3 | 0,0000 | 0,0000 | 0,0000 | 0,5706 | 0,2047 |
| CXCL11 | 6373 | chemokine (C-X-C motif) ligand 11 | 0,0000 | 0,0000 | 0,0000 | 0,4064 | 0,7601 |
| EGF | 1950 | epidermal growth factor | 0,0000 | 0,0000 | 0,0000 | 0,1616 | 0,7103 |
| FGF2 | 2247 | fibroblast growth factor 2 (basic) | 0,0000 | 0,0000 | 0,0000 | 0,9161 | 0,0085 |
| FGF6 | 2251 | fibroblast growth factor 6 | 0,0000 | 0,0000 | 0,0000 | 0,9457 | 0,1143 |
| FGF7 | 2252 | fibroblast growth factor 7 | 0,0000 | 0,0000 | 0,0000 | 0,4209 | 0,1055 |
| HGF | 3082 | hepatocyte growth factor | 0,0000 | 0,0000 | 0,0000 | 0,2634 | 0,3625 |
| IFNG | 3458 | interferon, gamma | 0,0000 | 0,0000 | 0,0000 | 0,4865 | 0,2673 |
| IGF1 | 3479 | insulin-like growth factor 1 | 0,0000 | 0,0000 | 0,0000 | 0,8533 | 0,1309 |
| IGFBP1 | 3484 | insulin-like growth factor binding protein 1 | 0,0000 | 0,0000 | 0,0000 | 0,1941 | 0,8371 |
| IGFBP2 | 3485 | insulin-like growth factor binding protein 2 | 0,0000 | 0,0000 | 0,0000 | 0,7740 | 0,1679 |
| IGFBP3 | 3486 | insulin-like growth factor binding protein 3 | 0,0000 | 0,0000 | 0,0000 | 0,7969 | 0,3447 |
| IGFBP4 | 3487 | insulin-like growth factor binding protein 4 | 0,0000 | 0,0000 | 0,0000 | 0,4793 | 0,3504 |
| IGFBP5 | 3488 | insulin-like growth factor binding protein 5 | 0,0000 | 0,0000 | 0,0000 | 0,5788 | 0,7259 |
| IL1B | 3553 | interleukin 1, beta | 0,0000 | 0,0000 | 0,0000 | 0,5769 | 0,3849 |
| IL4 | 3565 | interleukin 4 | 0,0000 | 0,0000 | 0,0000 | 0,5776 | 0,2225 |
| IL6 | 3569 | interleukin 6 | 0,0000 | 0,0000 | 0,0000 | 0,5367 | 0,1581 |
| IL13 | 3596 | interleukin 13 | 0,0000 | 0,0000 | 0,0000 | 0,1688 | 0,4807 |
| IL18BP | 10068 | interleukin 18 binding protein | 0,0000 | 0,0000 | 0,0000 | 0,4386 | 0,0922 |
| LEP | 3952 | leptin | 0,0000 | 0,0000 | 0,0000 | 0,3960 | 0,0115 |
| MMP1 | 4312 | matrix metallopeptidase 1 | 0,0001 | 0,0000 | 0,6050 | 0,1204 | 0,4489 |
| MMP2 | 4313 | matrix metallopeptidase 2 | 0,0000 | 0,2826 | 1,0000 | 0,9072 | 0,3142 |
| MMP3 | 4314 | matrix metallopeptidase 3 | 0,0000 | 0,0000 | 0,0000 | 0,2259 | 0,1787 |
| MMP8 | 4317 | matrix metallopeptidase 8 | 0,0001 | 0,0000 | 0,9476 | 0,3128 | 0,2530 |
| MMP9 | 4318 | matrix metallopeptidase 9 | 0,0000 | 0,0000 | 0,0000 | 0,1029 | 0,3370 |
| MMP10 | 4319 | matrix metallopeptidase 10 | 0,0032 | 0,0002 | 0,0000 | 0,1553 | 0,3733 |
| PPBP | 5473 | pro-platelet basic protein, CXCL7, NAP2 | 0,0000 | 0,0000 | 0,0000 | 0,9139 | 0,0044 |
| TIMP1 | 7076 | TIMP metallopeptidase inhibitor 1 | 0,0000 | 0,0000 | 0,6524 | 0,1088 | 0,2685 |
| TIMP2 | 7077 | TIMP metallopeptidase inhibitor 2 | 0,0073 | 0,8818 | 0,0433 | 0,1900 | 0,1768 |
| TIMP4 | 7079 | TIMP metallopeptidase inhibitor 4 | 0,0000 | 0,0000 | 0,0000 | 0,1117 | 0,1384 |
| TNF | 7124 | tumor necrosis factor | 0,0000 | 0,0000 | 0,2715 | 0,3083 | 0,5042 |
| TNFRSF11B | 4982 | tumor necrosis factor receptor superfamily, member 11b, OPG | 0,0000 | 0,4638 | 0,2813 | 0,7650 | 0,1704 |
| TNFRSF18 | 8784 | tumor necrosis factor receptor superfamily, member 18 | 0,0000 | 0,0000 | 0,8981 | 0,2321 | 0,2532 |
| VEGFA | 7422 | vascular endothelial growth factor A | 0,0000 | 0,0000 | 0,0000 | 0,4252 | 0,1142 |
